# Detecting and Accommodating Outliers in Meta-Analysis for Evaluating Effect of Albendazole on *Ascaris lumbricoides Infection*

**DOI:** 10.5812/ircmj.17648

**Published:** 2014-05-05

**Authors:** Hamid Alavi Majd, Khadijeh Najafi Ghobadi, Alireza Akbarzadeh Baghban, Nayebali Ahmadi, Elham Sajjadi

**Affiliations:** 1 Department of Biostatistics, School of Paramedical Sciences, Shahid Beheshti University of Medical Sciences, Tehran, IR Iran; 2 Department of Basic Sciences, School of Rehabilitation Sciences, Shahid Beheshti University of Medical Sciences, Tehran, IR Iran; 3 Department of Lab Sciences and Proteomics Research Center, School of Paramedical Sciences, Shahid Beheshti University of Medical Sciences, Tehran, IR Iran; 4 Department of Hematology, Tarbiat Modares University, Tehran, IR Iran

**Keywords:** Meta-Analysis, Albendazole, Outliers, Random Effects Variance Shift Model, *Ascaris lumbricoides*

## Abstract

**Background:**

Meta-analysis is a statistical technique in which the results of two or more independent studies, with similar objectives, are mathematically combined in order to improve the reliability of the results. The outliers, which may exist even in random models, can affect the validity and strength of meta-analysis results.

**Objectives:**

The current study uses "random effects variance shift model" to evaluate and correct the outliers in performing a meta-analysis study of the effect of albendazole in treating patients with *Ascaris lumbricoides *infection.

**Patients and Methods:**

The study used data from 14 clinical trials; each article was composed of two groups, a treatment group and a placebo group. These articles compared the effect of single dose intakes of 400 mg albendazole in treating two groups of patients with *Ascaris lumbricoides *infection. The articles were published in a number of internationally indexed journals between 1983 to 2013. For both groups in each article, the total number of participants, the number of those with *Ascaris lumbricoides *infection*,* and the number of those recovered following the intake of albendazole were identified and recorded. The relative risk (RR) and variance were computed for each article individually. Then, using meta-analysis, the RR was computed for all the articles together. In order to detect outliers the "random effects variance shift model" and "likelihood ratio test" (LRT) were used. Adopting the bootstrap method, the accuracy rates for sampling distribution of the tests, which were used for multiple testing, were obtained and the relevant graphs were depicted. For data analysis, STATA and R software were used.

**Results:**

According to meta-analysis results, the estimate for RR was 2.91, with a 95% confidence interval of 2.6 to 3.25. According to the method used in this study, three articles (articles number 4, 7, and 12) were outliers and, as such, they were detected in the graphs.

**Conclusions:**

We can detect and accommodate outliers in meta-analysis by using random effects variance shift model and likelihood ratio test.

## 1. Background

In health and medical sciences, the same topic may be investigated in numerous studies that may sometimes reveal contradictory results. One way of achieving a conclusive result is the formulation of a meta-analysis study, which mathematically combines and analyses the results of different studies to achieve a more reliable outcome. In this method, the results from a number of individual articles with similar objectives are combined and then are subjected to more conclusive statistical analyses (1). The main objective of meta-analysis is the application of a test with a statistical power greater than that of the individual studies. Nonetheless, it is essential to pay due attention to factors that may systematically affect the overall results when commenting on the validity and strength of the results in a meta-analytic computation. One of the factors that can negatively affect the validity of results in a meta-analysis computation is the presence of outlier, an element of a data set that distinctly stands out from the rest of the data. The most thorough method of identifying distant data in terms of outliers has been formulated by Hedges and Olkin (2). Numerous graphic methods have been introduced for the evaluation of unusual samples, but these methods can just be used for fix models (3, 4). However, bearing in mind that there are mixed models and random models as well, it is better to investigate outliers through these models too (5, 6). Gumedze and Jackson (7) introduced methods of detecting and accommodating outliers in a meta-analysis work by a random effects variance shift model. Therefore, the current study uses Gumedze and Jackson's model to measure the degree or size of outliers in a meta-analysis of the effect of albendazole on patients with *Ascaris lumbricoides *infection. *Ascaris lumbricoides* is one of the most soil-transmitted helminthes (STH) in the world. It is estimated that 4.5 billion individuals are at risk of STH infection (Ascaris lumbricoides, hookworms, and Trichuristrichiura) and as many as 1.2 billion individuals might be infected with Ascarislumbricoides, with *Ascaris lumbricoides*, close to 800 million with *Trichuris trichiura*, and more than 700 million with hookworms (8, 9). The majority of STH infected individuals are children and the infections are an important factor contributing to malnutrition in this age group (10).

## 2. Objectives

The current study used "random effects variance shift model" to evaluate and correct the outliers in performing a meta-analysis study of the effect of albendazole in treating patients with *Ascaris lumbricoides *infection.

## 3. Patients and Methods

The current study used data from 14 clinical trial articles, investigating the effect of albendazole in treating patients with *Ascaris lumbricoides* infection (11-24). The articles had already been published in internationally referenced journals from 1983 to 2013. The articles were first obtained through different sources like the internet, data banks, and internationally recognized journals with some special criteria indicated below and then were subjected to the relevant meta-analyses. We used the terms “albendazole” in combination with “trial” or “study”, “ascariasis”, and “*Ascaris lumbricoides”*. Bibliographies of identified articles were screened for additional relevant studies. The other criteria such as sample size, age, diagnostic method, and dosage were checked in selected articles. The patients under study had been matched in terms of age in the studies under meta-analysis. Besides, all the studies had used a similar definition for recovery, the same amount and frequency of albendazole (a daily single dose of 400 mg of oral medication), and a similar binary response variable for recovery versus nonrecovery. For each of the 14 articles, the total number of participants, the number of those infected with *Ascaris lumbricoides* as well as those recovered following the intake of albendazole (for each of the two groups), the effect size, and variance of the intervention were computed. Bearing in mind that each study was composed of both the albendazole and the placebo groups, the responses produced would follow a dichotomous variable. To compare the effect of albendazole on *Ascaris lumbricoides*, the cure rates were used to compare two groups under study. The priority index of the effect of albendazole as cure ratio in intervention group to the placebo group was considered the relative risk (RR). The effect size or RR is shown by Ɵ; then test statistic has to be defined for the significance of the effect size. Test statistics is defined by Q = Σ W_i_ (y_i_ − Ɵˆ)^ 2^ in which W_i_ = 1 / V_i_ and Ɵˆ = Σ (W_i_Y_i_) / Σ (W_i_). Under the null hypothesis, for all the effect sizes, which were similar or symmetrical, the distribution of Q statistics was chi-square with K-1 degree of freedom (25). To detect outliers in the data of current study, the random effect Variance Shift Outlier Model was used. As such, this method was used to detect and test the outliers. For meta-analysis, the STATA software was used and the R software was employed to administer this method (26). A brief account of the method is provided below.

### 3.1. Random Effect Variance Shift Outlier Model (RVSOM)

Following Gumedze and Jackson (7), basic model on standard random effects for meta-analysis is as follows:

*y* = µ1_*n*_ + *u* + *e *(1)

Where *y* is a *n*-vector of estimated treatment effects for the *n* independent studies, µ is the unknown overall treatment effect, 1_n_, is a *n*-vector of ones, u is a *n*-vector of unknown random effects, u ~ N (0,τ^2^ I_n_) where τ^2^ is the between-study variance, which is unknown, *e* represents residual errors with, e ~ N (0,R) where R = diag (δ^ 2^_1_, δ^ 2^_2,_…, δ^ 2^_n_). The elements of R, the study variances, are regarded as known. The variance-covariance matrix of (1) can then be written as *var*(y) = V = τ^2^I_n_ + R with the variance of the *i *th study treatment effect given as var(y_i_) = τ^2^ + δ^2^_i_.

### 3.2. Extending the Random Effects Model to the RVSOM

According to Gumedze and Jackson (7), the random effects variance shift outlier model (RVSOM) for the *i*th study (which allows an inflated variance for the *i*th study) takes the form:

Y = µ1_n_ + δ_j_d_j_ + u + e (2).

This adds an extra term δ_j_d_j_ to model (1), where d_j_ is the *j*th unit vector of length *n*, i.e. with value 1 in the *i*th position and zero elsewhere, and is an unknown random coefficient with δ_j_ ~ N (0,ω^2^_j_) for ω^2^_j_≥ 0.

The subscript *j* indicates which study has an inflated variance. Model (2) has the form of a simple linear mixed model with δ_j_ as a random effect with variance ω^2^j. The variance-covariance matrix for the data under the RVSOM for the jth observation is:

var (y) = ω^2^_j_ d_j_ d^΄^_j_ + V

An extension of model (2), which allows different inflated variances for more than one study, can be written as:

Y = µ1_n_ + D_I_ δ_I_ + u + e

Where I is a subset {1, 2, …, *r*} of studies considered to be outliers, D = [d_j_] is an *n* × *r* matrix containing entires of 0 and 1, where an entry of 1 in the ith row and jth column indicates that study *i* has the jth of *r* inflated variances, and δ_I_ is a *r* × 1 vector of unknown random effects. We referred to this model as an ‘extended RVSOM’ (7).

### 3.3 Administering the Random Effect Variance Shift Outlier Model

At First, we used forest plot diagram to detect outliers in our data, then we entered the outliers detected in forest plot in the RVSOM Model as the jth observation. Then the model was fitted to the data and the degree of ωˆ^ 2^_j_ for the jth was computed; the larger size of ωˆ^ 2^_j_, the more likely for it to detect as an outlier. The likelihood ratio test (LRT) was used to measure the size or magnitude of ω^ˆ 2^_j_.

The null hypothesis was H_0_:ω^ 2^_j_ = 0 against the alternative hypothesis was H_A(j)_:ω^2^_j_ > 0 for a RVSOM for observation *j*. Stram and Lee (27, 28) showed that the asymptotic null distribution of the test statistic for testing this type of hypothesis was a mixture of two chi-squared distributions on zero and one degree of freedom. However, Gumedze and Jackson (7) showed that for the RVSOM conditions it cannot be met; hence, following Gumedze et al. (29), we had to use a parametric bootstrap procedure to obtain the distribution of our test statistic.

### 3.4. Empirical Distribution of the LRT Statistic and Multiple Testing

Under the null hypothesis, when there are no outliers in the data, empirical distribution of the likelihood ratio test statistics by a parametric bootstrap procedure is as follow:

Step 1. Fit the null model (1) to the data to obtain estimates μ ^ and τˆ^2^.
Step 2. Generate a new data vector from model (1) and estimates μ ^ and τˆ^2^.
Step 3. Compute the likelihood ratio test statistics LRT j, j = 1,..., *n*, by fitting the model (2) to the simulated data and compute and save the order statistics of the set LRT j for j = 1,..., *n*.
Step 4. Repeat steps 2 and 3 *R* times. This step generates an empirical distribution of size *R* for each order statistic.
Step 5. Calculate the 100 (1-α)th percentile for each order statistic for the required significance level *α*. The percentiles, using α = 0.05 and k = 1 for largest order statistic, k = 2 for second largest order statistic, are shown in the plots given in the results.

## 4. Results

**Figure 1.**
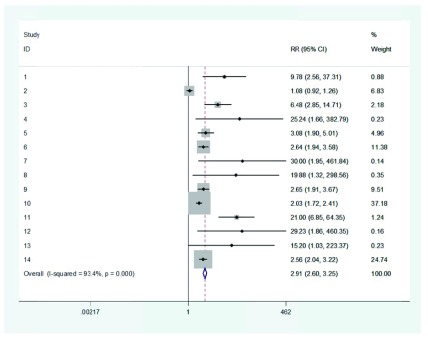
Forest Plot Diagram Used to Investigate the Effect of Albendazole on Patients With Ascaris lumbricoides Infection

**Figure 2.**
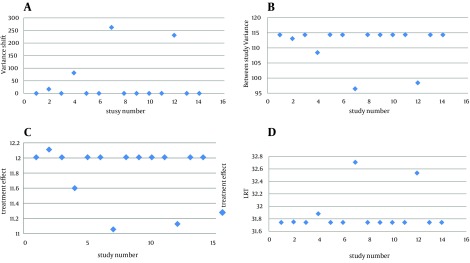
Random Effect Variance Shift Outlier Model Statistics Plotted Against Study Number for the Effect of Albendazole on Patients With *Ascaris lumbricoides* Infection (2a) Variance shift estimates.ω^2^_j_; (2b) random effect variance estimates τˆ^2^; (2c) estimates for treatment effect µˆ; (2d) likelihood ratio test statistics for a RVSOM for study j, plotted against study number.

The data used in this study were taken from 14 clinical trial articles that investigated the effect of albendazole on patients with *Ascaris lumbricoides *infection; y_i_ indicated the relative risk in the *i*th article. As the forest plot diagram (Figure 1) indicated, articles 4, 7 and 12 were different from the rest of studies. The results of RVSOM model are shown in Figure 2 and Table 1.

Figure 2a shows the estimates ω^2^_j_ form the jth RVSOM and the next two plots, Figures 2b and 2c, show the corresponding estimates of the between study variance and the treatment effect. The plot 2d shows the likelihood ratio statistics from which we see that observations 4, 7 and 12 are clearly detected as expected outliers; in particular, its LRT statistic is around three times the threshold for the first order statistic. All these figures refer to the fact that articles 4, 7 and 12 had served as outliers.

Table 1 includes the information of the articles used for meta-analysis, containing the names of the authors, year of publication, and the number of patients used albendazole regardless of its effect.

**Table 1.** Results From Clinical Trial Articles Investigating the Effect of Albendazole on Patients With *Ascaris lumbricoides* Infection (Al)^a^ (11-24)

Trial	Authors	Year	Albendazole		Placebo	
			A1^+^	A1^-^	A1^+^	A1^-^
**1**	Oyediran and Oyejide	1983	22	5	2	22
**2**	El-Masry et al.	1983	11	0	36	4
**3**	Bwibo and Pamba	1984	36	4	5	31
**4**	Ovedoff	1984	16	0	0	12
**5**	Chien et al.	1989	37	4	12	29
**6**	Upatham et al	1989	74	4	27	48
**7**	Stephenson et al.	1990	7	0	0	15
**8**	Sinniah et al.	1990	51	5	0	10
**9**	Beach et al.	1999	61	1	23	39
**10**	Olds et al	1999	179	40	92	137
**11**	Patrick P et al.	2009	63	25	3	85
**12**	J. Ndibazza et al.	2010	9	3	0	19
**13**	Speich B et al.	2012	9	0	0	7
**14**	Wiria AE et al.	2013	144	65	64	174

^a^ A1^+^ refers to those recovered following the treatment and A.1^-^ refers to those who did not recover following the treatment.

Table 2 shows that the inferences were very robust when these three outliers were down weighted using the extended RVSOM (which includes three ω^2^_j_, j = 4, 7, and 12 terms in a single model) and our findings greatly alleviated any concerns about the potential impact of outliers in these data. Once again, our proposed methodology performed well.

**Table 2.** Estimated Parameters for Models Fitted to Investigate the Effect of Albendazole on Patients With *Ascaris lumbricoides* Infection ^a^

-	Model M_ 0_		Model M_ 1_	
**Parameter**	**Estimation**	**95% CI**	**Estimation**	**95% CI**
**μ**	11.247376	(8.8,13.7)	10.29457445	(5.16, 15.43)
**τ^2^**	20.083981	-	85.23284239	-
**ω_4_^2^**	-	-	140.53558626	-
**ω_7_^2^**	-	-	302.27428826	-
**ω_12_^2^**	-	-	0.01746554	-

^a^ Lumbricoides data: Overall treatment effect (µ), Variance shift estimates for *j*th the study (ω^2^_j_), and between-study variance (τ^2^). M_ 0_: Random effects; model M _1_: Extended RVSOM for study 4,7, and 12.

## 5. Discussion

The results obtained from meta-analytic evaluation of 14 studies investigating the effect of albendazole on patients with *Ascaris lumbricoides*, detected articles 4, 7, and 12 as outliers. The articles under study were published in the internationally indexed journals during 1983 to 2013. The study used RVSOM to detect outliers. The results obtained from the tests and the relevant figures might indicate that the studies in rows 4, 7, and 12 served as outliers in this meta-analytic review and that the findings were largely reliable. Of course, with application of the method used in article 30, the articles 2, 10, and 14 were also detected as outliers. In this regard, in another research, a meta-analysis study was conducted to investigate the effect of albendazole on two groups of case and control patients afflicted with *Trichuris trichiura*. The results indicated a relative risk estimate of 2.06 with 95% confidence intervals of 2.4 to 1.76. Therefore, with such results in mind, it can be claimed that, the cure ratio of individuals infected with *Trichuris trichiura* using albendazole was two times more than those who did not use the drug (22-24, 29, 30). In the current study, however, the relative risk estimate was 2.91 with 95% confidence interval of 2.6 to 3.25, indicating that the ratio of individuals infected with *Ascaris lumbricoides* using albendazole was nearly three times more than those who did not take the drug. Therefore, it can be concluded that albendazole has been more effective in treating *Ascaris lumbricoides* than curing *Trichuris trichiura*.
